# Wellbeing and quality of life secondary outcomes from a Mediterranean Diet and walking randomised controlled trial in older Australians

**DOI:** 10.1017/S1368980026102274

**Published:** 2026-03-25

**Authors:** Ella L. Bracci, Courtney R. Davis, Denny Meyer, Michael Kingsley, Jeff Breckon, Catherine Itsiopoulos, Helen Macpherson, Kade Davison, Andrew Scholey, Greg Kennedy, Leonie Segal, Andrew Pipingas, Karen J. Murphy

**Affiliations:** 1 Clinical and Health Sciences, University of South Australia, Adelaide, South Australia, Australia; 2 Justice and Society, University of South Australia, Adelaide, South Australia, Australia; 3 Centre for Mental Health and Brain Sciences, Swinburne University of Technology, Melbourne, Victoria, Australia; 4 Department of Exercise Sciences, The University of Auckland, Auckland, New Zealand; 5 Holsworth Research Initiative, La Trobe University, Bendigo, Victoria, Australia; 6 School of Health and Life Sciences, Teesside University, Middlesbrough, UK; 7 School of Health and Biomedical Sciences, RMIT University, Bundoora, Victoria, Australia; 8 Institute for Physical Activity and Nutrition, Deakin University, Burwood, Victoria, Australia; 9 Allied Health and Human Performance, University of South Australia, Adelaide, South Australia, Australia; 10 Nutrition Dietetics and Food, Monash University, Notting Hill, Victoria, Australia; 11 Health Economics and Social Policy Group, University of South Australia, Adelaide, South Australia, Australia

**Keywords:** Mediterranean Diet, Wellbeing, Quality of life, Psychological health

## Abstract

**Objective:**

The Mediterranean Diet (MedDiet) and physical activity (PA) can enhance mood and support psychological wellbeing in adults. However, the combined effect is relatively unknown. MedWalk aimed to determine the combined effect on wellbeing, psychological health and quality of life (QoL), compared to a control group.

**Design:**

This is an analysis of secondary outcomes from the MedWalk 12-month cluster-randomised controlled trial. Participants completed the Total and Secure Flourishing Index (FI), the four domain General Health Questionnaire (GHQ-28) and the 8-domain Assessment of Quality of Life (AQoL-8D). Data were analysed using general linear models using change scores (FI and AQoL-8D) or generalised linear mixed models with a time × group interaction effect (GHQ-28).

**Setting:**

Independent living facilities across South Australia and Victoria in 2021–2022.

**Participants:**

One hundred and sixty-one older men and women.

**Results:**

Participants were 74·9 ± 5·9 years of age and predominantly female (74 %). A greater improvement was found for the MedWalk group (marginal means (MM) = 1·65, se = 1·36) than the control group (MM = –2·50, se = 1·32) for the Total Flourish score (*P* = 0·003) and Secure Flourish score (*P* = 0·009) ((MM = 1·06, se = 1·65) *v*. (MM = –3·34, se = 1·61)) from baseline to 6 months. The MedWalk group (MM = 0·021, se = 0·014) had more positive changes (*P* = 0·048) to the Mental Health AQoL-8D domain than the control group (MM = –0·007, se = 0·014). No significant group × time interactions were identified for the GHQ-28.

**Conclusions:**

Combined MedDiet and walking interventions can modify psychological health, wellbeing and QoL in relatively healthy populations.

Higher rates of poor mental health, social isolation, depression and co-morbidity exist in older adults with one in eight adults reporting high or very high levels of psychological distress during the 2017–2018 National Health Survey in Australia^(1)^. Factors influencing these high rates may include reduced mobility, poor physical and emotional health and social isolation, or lack of social connectedness^(2)^. These factors may also be further exacerbated by chronic disease and/or multimorbidity or polypharmacy. Interventions that target multiple lifestyle domains such as emotional and social health, in addition to physical health, are therefore warranted.

Emotional health and wellbeing can be described and referred to as broad multidimensional constructs, including the hedonic and eudemonic aspects of happiness, life satisfaction, and meaning and purpose (Figure 1). Additionally, wellbeing encompasses physical, social and spiritual health. Meanwhile, psychological health is a mental state and reflects symptoms of emotional and cognitive functioning (i.e. anxiety, insomnia, low mood and social dysfunction). In contrast, quality of life (QoL) is the perceived impact of health on various life and lifestyle domains such as physical, emotional and social functioning (Figure 1).

**Figure 1.**
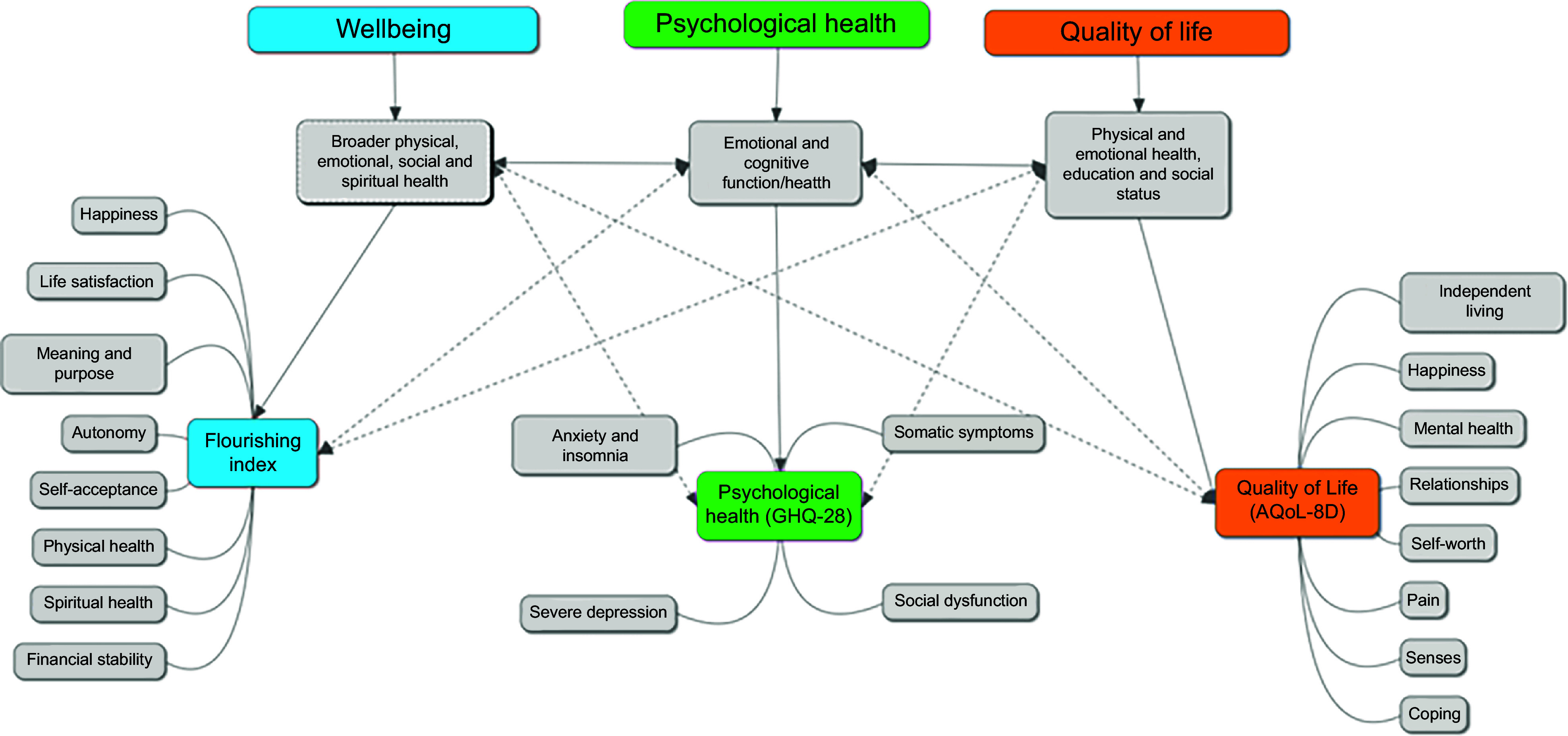
Overview of wellbeing, including flourishing, psychological health and quality of life concepts.

Previous research suggests that a higher quality diet, that is, a diet that includes a variety of foods from the five core food groups, is significantly associated with better QoL, mood and wellbeing, including ‘flourishing’^(3,4)^. While flourishing is not necessarily equivalent to wellbeing or psychological health, it is a holistic framework from positive psychology that reflects the optimal function across multiple lifestyle domains that are encompassed and overlap with wellbeing as a concept^(5–7)^.

Despite the role of diet in psychosocial health, Australians typically report following a ‘Western’-style diet which has poor diet quality characterised by high intake of discretionary or ultra-processed foods and high intakes of saturated fat and added salt and sugar^(8–11)^. This trend of consumption has similarly been reported in other countries such as America and the United Kingdom. In contrast, ‘higher quality’ dietary patterns including high intake of fruits and vegetables, fish and wholegrains, and foods high in magnesium, *n*-3 long-chain PUFA, vitamin C and flavanols have been suggested to improve mood and wellbeing^(12–14)^.

The Mediterranean Diet (MedDiet) is a predominantly plant-based diet characterised by the intake of extra virgin olive oil as the main culinary fat, leafy green vegetables, fresh fruit, legumes, nuts, seeds, wholegrain breads and cereals, fermented dairy foods, fish and smaller portions of red meat^(15)^. Meta-analyses from observational studies have demonstrated a lower risk of depression and better mental health outcomes when following a MedDiet^(16)^. Further, results from a number of recent Australian MedDiet RCT have also indicated improvements to other measures of psychosocial health such as mood and QoL^(17–21)^. For example, 6-month dietary interventions such as the LIILAC study^(20)^ and HELFIMED^(21,22)^ showed significant mood improvements compared to control or social comparator groups^(20)^. LIILAC was a 6-month MedDiet and exercise intervention in independent living participants aged 60–90 years^(20)^ where both the combined MedDiet and exercise group and exercise-only group led to improvements in emotional state as assessed by the Depression Anxiety Stress Scale (DASS) (*P* < 0·05)^(20)^. However, there was no change in mood as measured by the Profile of Mood States (POMS)^(20)^.

Similarly, physical activity (PA) can contribute to better mood and psychological health and improve QoL^(16,23)^. However, Australians typically lead lifestyles characterised by prolonged sedentary behaviours and limited physical activity (PA). Walking is the most common form of aerobic exercise, is a relatively low burden for older populations and helps to prevent frailty, muscle loss and improve bone strength and mobility^(24,25)^. At a population level, walking groups or interventions with targeted walking protocols can provide an opportunity to encourage wider adoption of PA^(26–29)^. A 2015 systematic review and meta-analysis with forty-two included studies and almost 2000 participants identified a range of health benefits from walking groups^(30)^. Namely, there were statistically significant improvements in blood pressure and heart rate (*P* < 0·001), body fat and BMI (*P* = 0·001, *P* = 0·003, respectively) and QoL as measured by the SF-36 (*P* = 0·003)^(30)^. This ‘group-based’ approach also provides a level of accountability and provision of social connectedness which aligns with healthy lifestyle patterns and pillars of lifestyle medicine.

In summary, interventions that encompass all six pillars of lifestyle medicine (nutrition, PA, stress management, substance avoidance (tobacco and excessive alcohol), restorative sleep and social connection))^(31)^ can improve psychological health and wellbeing for populations with and without depression. As these outcomes can be modified in response to interventions, there is potential to measure and evaluate the role of ‘flourishing’ behaviours and attributes that relate to wellbeing and psychosocial health. These outcomes have not been fully explored in large-scale RCT in older independent living populations who may be at higher risk of social isolation, depression, poor wellbeing and decreased QoL. Further, the potential for a synergistic effect when combining a MedDiet with exercise needs to be further explored > 6 months.

## Aims

The primary aim of this study was to address the identified gaps and determine the effect of a combined 12-month MedDiet and walking intervention on wellbeing, including flourishing, psychological health and QoL from three self-reported questionnaires: the Flourishing Index (FI), the General Health Questionnaire (GHQ-28) and the Assessment of Quality of Life (AQoL-8D).

## Methods

### Participants

This is an analysis of secondary outcomes from the MedWalk trial. The protocol, including statistical analysis plan for MedWalk, has been previously published^(32)^. In brief, MedWalk was a multi-site cluster-randomised controlled trial in independently living older South Australians and Victorians. Rolling recruitment occurred during July to September 2021 and was predominantly from independent living facilities with some recruitment occurring from the community in Victoria^(32)^.

**Figure 2.**
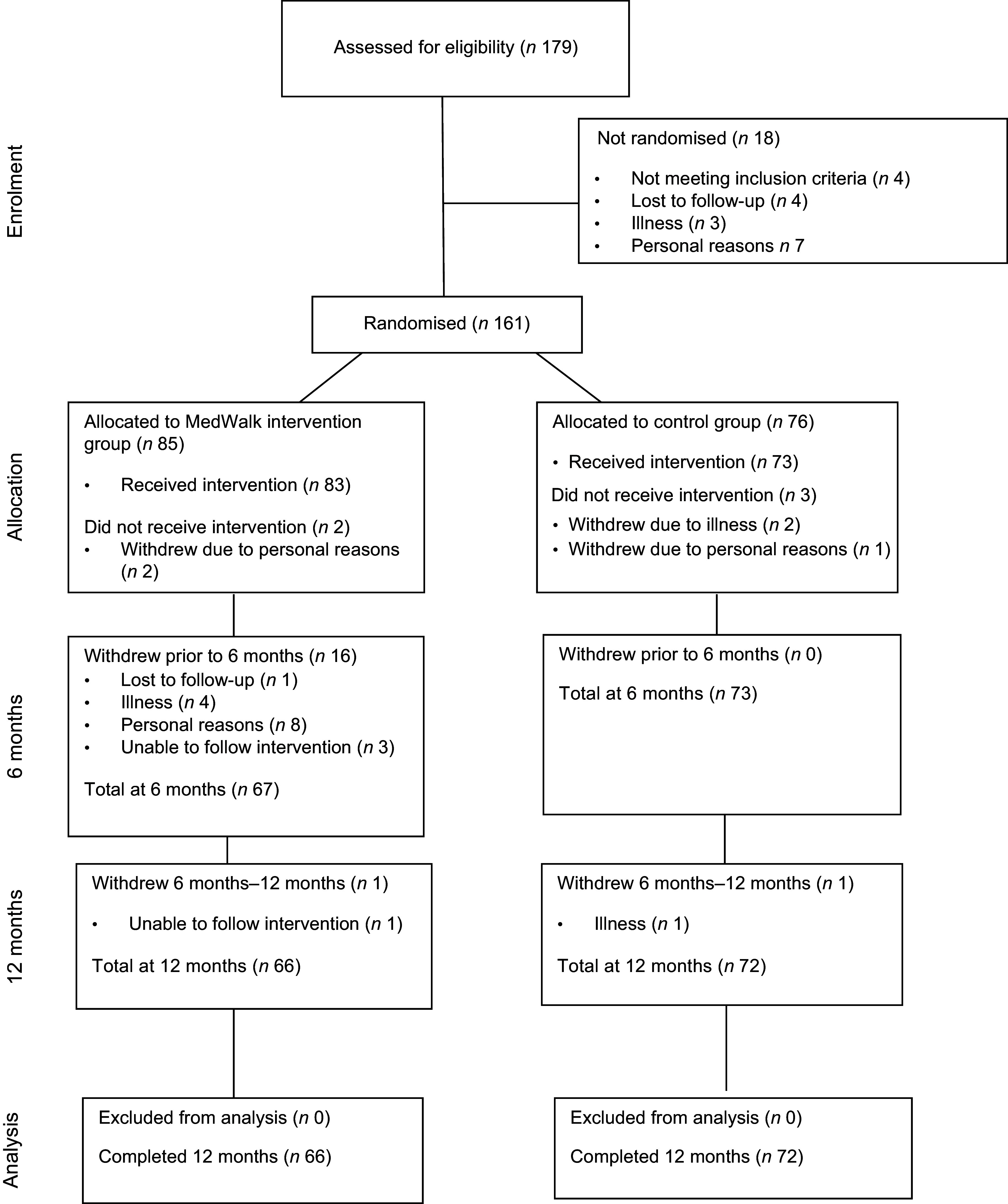
MedWalk participant CONSORT diagram.

Participants were stratified by demographics (i.e. age, self-reported gender identity and BMI) into either intervention (MedWalk) or control. Each site (independent living facility or community group) was cluster-randomised, with participants residing in the same facility following the same intervention, either the MedDiet and walking intervention, or control. Community groups were recruited based on the same inclusion criteria and clustered based on demographic commonalities to ensure homogeneity for allocation purposes^(32)^. Stratification was performed by an independent statistician who was blinded to the intervention code and did not partake in data collection. Randomisation was revealed to participants by blinded research staff after the baseline assessment^(32)^. A total of 161 participants commenced the study, eighty-five of whom were intervention and seventy-six control (Figure 2). Baseline assessments were completed by October 2022.

### MedWalk inclusion and exclusion criteria

The primary inclusion criteria included participants aged 60–90 years and living independently in South Australia or Victoria, mostly in ‘retirement villages’, also referred to as independent living facilities (Table 1). A small proportion of the Victorian cohort was recruited from the community due to the impact of the COVID-19 pandemic^(32)^.

**Table 1.** Inclusion and exclusion criteria for the MedWalk intervention as modified from the protocol paper^(32)^

Inclusion	Exclusion
Aged 60–90 years Residing in an independent living facility Capable of walking independently Fluent in written and spoken English Adequate vision and hearing Willing to provide samples (blood and faecal) Able to participate in regular scheduled visits over 12-month period	MMSE below 24 (considered cognitive impairment) Pre-existing diagnosis of mental health condition OR > 10 on Geriatric Depression Scale (GDS) Previous history of stroke or neurological conditions Use of cholinesterase inhibitors Diagnosed food allergy or intolerance (nuts/seafood/EVOO) Meets physical activity (PA) guidelines (> 150 min of moderate-to-vigorous PA) and has high adherence to a MedDiet (> 10 on PREDIMED questionnaire)

MMSE, Mini Mental State Evaluation; EVOO, extra virgin olive oil; PREDIMED, ‘Prevención con Dieta Mediterránea’ study which aimed to determine long-term effects of a MedDiet on CVD.

### MedWalk intervention

The MedWalk study had twenty-one clusters and was conducted in seventeen retirement villages. The remaining clusters underwent testing at a university site due to being recruited from the community^(32)^. The primary aim of the MedWalk intervention was to reduce cognitive decline with the primary outcome 12-month change in paired associate learning from the Cambridge Neuropsychological Test Automated Battery (CANTAB). Sample size and statistical powering are described elsewhere. A range of secondary outcomes including cognition and other CANTAB tasks, mood, sleep, QoL, wellbeing, psychological health, blood biomarkers, cardiovascular health, frailty, Mediterranean Diet Adherence and PA were also assessed at either baseline and 12 months, or during the three major data collection visits (0, 6 and 12 months) using self-reported tools as described in the protocol paper^(32)^.

Participants in the intervention group received dietetic support from study dietitians to assist with following a MedDiet and participated in walking groups, led by an exercise physiologist^(32)^. The MedDiet component was based on modified PREDIMED dietary guidelines and tailored to each participant to encourage long-term adoption. Participants also received a MedDiet recipe book, food hampers (fortnightly, followed by monthly) with MedDiet foods (extra virgin olive oil, wholegrains, canned fish and legumes and dried herbs) and meal planning and recipe information. The walking component was delivered weekly for the first 6 months by exercise scientists and exercise physiologists and then reduced to monthly for the remainder of the trial.

Both dietitians and exercise physiologist were trained in Motivational Interviewing-Cognitive Behaviour therapy (MI-CBT) which was delivered as part of the intervention^(32)^. MI uses a combination of relational (evocation, compassion, acceptance and collaboration) and technical micro-skills (e.g. Open questions, Affirmations, Reflective listening, Summarising; OARS) to build client autonomy towards change, exploring motives and perceived barriers towards change^(33)^. The CBT components are action-orientated treatments including social support; identifying change barriers and goals; developing flexible goal setting; action planning; managing relapse; building maintenance strategies and processes for self-monitoring^(34)^.

The control group were instructed to maintain and follow their usual lifestyle and were provided with a total of AUD$150 as an honorarium over the study duration^(32)^.

### Demographic variables

a) Self-reported income data collected were collapsed into a binary variable with 1 for ‘Always sufficient income for needs’ and 0 for ‘Mostly, Sometimes or Rarely sufficient income for needs’.

b) Self-reported general health data collected were collapsed into a binary variable with 1 for ‘good or very good general health’ and 0 for ‘average, poor or very poor general health’.

## Measures

This analysis of secondary MedWalk outcomes explored different aspects of wellbeing, psychological health and QoL using a range of self-reported measures.

### The Flourishing Index

The FI developed at Harvard University is a valid and reliable (Cronbach’s *α* = 0·89 for Total Flourish and 0·86 for Secure Flourish) wellbeing questionnaire with five main constructs. Constructs measure ‘happiness and life satisfaction’, ‘physical and mental health’, ‘meaning and purpose’, ‘character and virtue’ and ‘close social relationships’ to create a Total Flourish score (maximum score = 100)^(5,7)^. Each construct has two questions with responses measured on a scale from 0 to 10 with a higher score having a more positive connotation with the question (i.e. strongly agree, completely true), whereas a lower score represents lower agreement (i.e. poor, strongly disagree, not true of me)^(5)^. The addition of the secure construct and two additional questions related to financial and material (housing, food) stability can be calculated with a maximum Secure Flourish score of 120 from the twelve questions. A higher score indicates greater wellbeing^(5)^. Further information on the translation, adaptations and psychometric properties is available (https://hfh.fas.harvard.edu/measuring-flourishing).

### The 8-domain Assessment of Quality of Life

The AQoL-8D is a valid and reliable (Cronbach’s *α* = 0·97) health-related QoL instrument developed at Monash University^(35,36)^. Dimensions of the AQoL-8D include relationships, independent living, mental health, coping, pain, senses, self-worth and happiness and comprise both physical and psychosocial constructs^(36)^. Participants respond to a series of thirty-five statements, typically providing a Likert-style scale of 5–6 response options. Scores from the eight domains and an additional total ‘utility’ score are calculated using the published algorithm (https://aqol.com.au/) and range from –0·4 (worse than death), 0 (death) to 1·0 (full health). The AQoL-8D is a tool for assessing health-related QoL with the utility score derived for economic analyses to determine quality-adjusted life-years^(36)^. The utility score analysis and associated cost-consequence from MedWalk are presented elsewhere^(37)^.

### The 28-item General Health Questionnaire

The General Health Questionnaire, developed in the late 1970s by psychiatrists^(38,39)^, is a reliable and valid (test–retest = 0·78–0·9^(40)^; Cronbach’s *α* = 0·9–0·95^(41)^) 28-question survey aimed at determining changes to mental state over the past few weeks to capture psychological health and identify individuals at risk of developing psychiatric disorders^(39)^. The GHQ-28 comprises four subscales including somatic symptoms, anxiety and insomnia, social dysfunction and severe depression and has a composite score. Questions relate to feeling run down, feeling panicky or scared, enjoyment of day-to-day activities and sleep^(39)^. Participants have the option of four responses with varying levels of agreement. A higher score is indicative of greater psychological distress (minimum score = 0, maximum score = 84)^(39)^.

### Statistical analyses

All statistical analyses were conducted using SPSS for Windows version 29 (IBM Corp. Released 2021. IBM SPSS Statistics for Windows, version 28·0. IBM Corp). The level of significance that was used for all hypothesis testing assumed a two-sided type 1 error rate (*P* < 0·05). Data were available at baseline, 6 months and 12 months for the FI and GHQ-28 and at baseline and 12 months for the AQoL-8D. However, there was attrition at 6 months when a small percentage of participants dropped out of the study (Figure 2). Incomplete data were not discarded, and missing data were not replaced or imputed. Differences in baseline characteristics between groups were determined using independent samples *t* tests for nominal variables and chi-square tests for categorical variables. A Bonferroni adjustment was used to test for differences in baseline PA as there were three measures for this variable.

A binary logistic regression analysis was used to identify factors associated with attrition. Group (MedWalk or control), state (South Australia or Victoria), self-reported gender identity, a binary variable for income meeting needs (0) or income not meeting needs (1) and health status coded as (0) for very good or good health and (1) for average, poor or very poor health from self-reported demographic questionnaires were included in group comparisons over time due to differences in the attrition rate and baseline characteristics between groups.

The AQoL-8D, FI and GHQ-28 data were tested for normality. Normality tests indicated that the AQoL-8D domains and the Flourish Index data breached assumptions as indicated by histograms, Q-Q plots and skewness and kurtosis. After transforming the data, data were still not normally distributed; therefore, change scores were used as these were found to be normally distributed.

Change scores for the Flourish Index and AQoL-8D domain scores were analysed through general linear models. A general linear model was used for each time period (i.e. baseline to 6 months, 6 months to 12 months and baseline to 12 months). Nominal variables, including group, state, self-reported gender identity, a binary variable for income meeting needs, and health status, were included as fixed factors. Age (years) and education (years) were included as covariates with no risk of multi-collinearity (*r* = –0·166 at baseline).

A gamma generalised linear mixed model was appropriate to compare the groups for changes over time in the raw GHQ-28 scores (by testing the time × group interaction effect) due to the positive skew in this distribution. As the GHQ-28 questionnaire includes ‘0’ as a value, +1 was added to the four domain scores to run the gamma generalised linear mixed model, but this was not needed for the composite score. Time, group, a time × group interaction effect, state, age, self-reported gender identity, education years and binary variables for health status and income meeting needs were included as fixed effects in the model. A random effect block with study ID and a random intercept was used.

## Results

### Participants

Characteristics of participants who completed cognitive testing (primary outcome) at baseline (*n* 157) have been published in the protocol paper^(32)^.

Baseline characteristics of the overall sample included in this analysis of secondary outcomes (*n* 161) are in Table 2. At baseline, 156 participants completed the FI, while 155 completed the AQoL-8D and GHQ-28. At baseline, most participants reported their health status as ‘very good’ or ‘good’ and had enough income to meet their needs.

**Table 2.** Characteristics of MedWalk participants (*n* 161)

	MedWalk (*n* 85)		Control (*n* 76)		All (*n* 161)	
	Mean	sd	Mean	sd	Mean	sd
Age, years	75·3	6·0	74·5	5·7	74·9	5·9
Education, years	15·5	4·2***	13·5	3·2***	14·5	3·8
	*n*	%	*n*	%	*n*	%
Gender identity						
Female	62	73	57	75	119	74
Male	23	27	19	25	42	26
Income status at baseline						
Always have enough	57	67	55	72	112	70
Do not always have enough	28	33	21	28	49	30
Health status at baseline						
Very good or good	72	85	66	87	138	86
Average, poor, very poor	13	15	10	13	23	14
MEDAS (14pt max)	5·7	1·9	5·6	2·0	5·6	2·0
Daily PA (IPAQ-E)						
Walking/d (min)	68	52	72	55	70	53
Moderate PA/d (min)	78	59	70	57	74	58
Vigorous PA/d (min)	13	32*	24	41*	18	37

MEDAS, Mediterranean Diet Adherence Screener; PA, Physical Activity; IPAQ-E, International Physical Activity Questionnaire modified for the elderly and based on the previous 7 d of activity.

*P < 0·05; ***P < 0·001.

Years of education differed significantly between groups (*P* < 0·001) as did PA, but the difference in mean minutes of vigorous PA per d (*P* = 0·030) was not significant when a Bonferroni adjustment was made for multiple testing.

#### Mean outcome scores

Mean raw scores for the FI (Total Flourish score and Secure Flourish score), AQoL-8D and GHQ-28 domains and composite score for change score data are in Additional File 1 (Table A1).

### Flourishing Index change score analysis – general linear models

#### Total Flourish

For Total Flourish changes from baseline to 6 months, there was a significant group difference (*P* = 0·003) with more positive changes for the MedWalk group (marginal means (MM) = 1·65, se = 1·36) than the control group (MM = –2·50, se = 1·32) (Table 3).

**Table 3.** Marginal means (MM) for change scores for the Flourishing Index and AQoL-8D

	MedWalk		Control		Group comparison^†^			
	MM	95 % CI lower, upper bound	MM	95 % CI lower, upper bound	F (1,df)	Error df	*P*-value	Partial *η* ^2^
Variable								
Total Flourish (baseline, 6 and 12 months)								
Baseline to 12 months	−2·17	–3·27, 2·83	**−**1·02	1·45, –3·90	0·294	124	0·589	0·002
6 months to 12 months	−1·14	–3·97, 1·70	1·77	–0·928, 4·46	4·32	124	0·040^*^	0·034
Baseline to 6 months	1·65	–1·04, 4·34	**−**2·50	–5·12, 0·112	9·30	122	0·003^**^	0·071
Secure Flourish (baseline, 6 and 12 months)								
Baseline to 12 months	−0·722	–4·03, 2·49	−1·50	–4·57, 1·57	0·212	124	0·646	0·002
6 months to 12 months	−0·836	–4·42, 2·74	2·04	–1·36, 5·44	2·68	124	0·104	0·021
Baseline to 6 months	1·06	–2·22, 4·33	−3·34	–6·52, –0·154	7·03	122	0·009^**^	0·054
AQoL-8D Domain								
Independent living baseline to 12 months	0·012	–0·020, 0·043	−0·013	–0·043, 0·018	2·36	125	0·127	0·019
Happiness baseline to 12 months	−0·025	–0·049, 0·000	−0·018	–0·042, 0·006	0·302	125	0·584	0·002
Mental Health baseline to 12 months	0·021	–0·007, 0·049	−0·007	–0·034, 0·020	3·97	125	0·048^*^	0·031
Relationships baseline to 12 months	−0·011	–0·047, 0·024	−0·013	–0·057, 0·021	0·011	125	0·915	0·000
Pain baseline to 12 months	0·018	–0·040, 0·076	0·000	–0·056, 0·055	0·408	124	0·524	0·003
Senses baseline to 12 months	0·013	–0·020, 0·046	−0·002	–0·034, 0·029	0·862	125	0·355	0·007
Coping baseline to 12 months	−0·028	–0·062, 0·005	−0·030	–0·062, 0·002	0·012	125	0·912	0·000

AQoL-8D, 8-domain Assessment of Quality of Life.

† Controling for state, gender identity, income, general health, age and education (refer Additional File Tables A2 and A3 for more details).

*P < 0·05, **P < 0·01.

Between 6 and 12 months, the control group had significantly more positive changes (*P* = 0·040) compared to the MedWalk group ((MM = 1·77, se = 1·36) *v*. (MM –1·14. se 1·43)), indicating a regression in scores for MedWalk participants (Table 3). Between 6 and 12 months, South Australians had more positive changes than Victorians (B coefficient = 3·23, *P* = 0·033) (Table A2, A3).

From baseline to 12 months, there were no significant group differences for Total Flourish scores between the MedWalk (MM = –2·17, se = 1·54) and control group (MM = –1·02, se = 1·45) (Table 3).

#### Secure Flourish

For changes in Secure Flourish scores from baseline to 6 months, there was a significant group difference (*P* = 0·048) with more positive changes for the Medwalk group (MM = 1·06, se = 1·65) than the control group (MM = –3·34, se = 1·61) (Table 3).

Between 6 months and 12 months, no significant group differences were observed for Secure Flourish scores between MedWalk (MM = –0·836, se = 1·81) and control groups (MM = 2·04, se = 1·72). Similarly, for Secure Flourish changes from baseline to 12 months, there was no significant group difference between MedWalk (MM = –0·772, se = 1·65) and control groups (MM = –1·50, se = 1·56) (Table 3).

### The 8-domain Assessment of Quality of Life change score analysis – general linear models

There were no significant group differences for changes between baseline and 12 months for the 8 AQoL domains, except for the Mental Health domain (*P* = 0·048) where the MedWalk group had more positive changes (MM = 0·021, se = 0·014) than the control group (MM = –0·007, se = 0·014) (Table 3).

Tests of between-subject effects indicated females had significantly less positive change for the Independent Living domain than males (B coefficient = –0·046, *P* = 0·010) (Table A2, A3). Having adequate income was associated with a significant positive change for the Relationship domain (B coefficient = 0·043, *P* = 0·027), as was reporting better health status (B coefficient = 0·053, *P* = 0·041) indicating greater improvements during the trial. Increasing age was associated with a significant positive change for the Senses domain (B coefficient = 0·005, *P* = 0·002) (Table A2, A3).

### The 28-item General Health Questionnaire - gamma generalised linear mixed models

No significant group × time interactions were identified for the composite GHQ-28 score or domain scores.

However, significant predictors of improvements to composite score and GHQ-28 domains were health status, age and state (Table A4). Better health status was associated with a decrease in the somatic symptoms (B coefficient = –0·452, *P* =< 0·001), anxiety and insomnia (B coefficient = –0·316, *P* = 0·024), social dysfunction (B coefficient = –0·122, *P* = 0·015), severe depression (B coefficient –0·329, *P* = 0·009) and composite GHQ-28 scores (*P* < 0·001) indicating less psychological distress (Table 4). South Australians also reported lower anxiety and insomnia domain scores (B coefficient = –0·563, *P* = 0·007) and composite GHQ-28 scores (B coefficient = –0·357, *P* = 0·014) compared to Victorians (Table A4). Increasing age was associated with greater social dysfunction (B coefficient = 0·010, *P* = 0·001) (Table A4).

**Table 4.** Marginal means (MM) for GHQ-28 domains and composite scores

	MedWalk		Control		Time × group interaction effect^†^		
	MM	95 % CI lower, upper bound	MM	95 % CI lower, upper bound	F(2,df)	df	*P*-value
GHQ-28 variables							
Somatic							
Baseline	4·51	3·81, 5·33	4·61	3·83, 5·54	1·78	416	0·170
6 months	4·37	3·64, 5·24	5·38	4·48, 6·46			
12 months	4·38	3·64, 5·23	5·29	4·40, 6·35			
Anxiety insomnia							
Baseline	3·95	3·28, 4·76	3·55	2·89, 4·35	2·59	415	0·076
6 months	3·35	2·74, 4·08	3·53	2·88, 4·33			
12 months	3·50	2·86, 4·28	3·95	3·22, 4·84			
Social dysfunction							
Baseline	7·80	7·29, 8·36	8·34	7·74, 8·99	0·822	415	0·440
6 months	7·62	7·07, 8·22	8·51	7·90, 9·17			
12 months	7·84	7·26, 8·46	8·25	7·66, 8·90			
Severe depression							
Baseline	1·57	1·33, 1·84	1·43	1·19, 1·71	1·67	415	0·189
6 months	1·40	1·17, 1·66	1·49	1·25, 1·78			
12 months	1·48	1·24, 1·76	1·45	1·21, 1·74			
Composite GHQ-28 score							
Baseline	14·5	12·9, 16·4	14·5	12·6, 16·6	2·41	415	0·091
6 months	13·5	11·8, 15·4	15·6	13·6, 17·9			
12 months	13·9	12·2, 15·9	15·5	13·6, 17·8			

GHQ, General Health Questionnaire.

† Controlling for state, gender identity, income, general health, age and education (refer Additional File Tables A4 for more details).

## Discussion

This study describes the impact of a 12-month MedDiet and walking intervention on wellbeing, QoL and psychological health in a relatively healthy independent living population whose perceived health prior to the intervention was ‘very good’ or ‘good’. This study did not seek to determine whether the MedDiet specifically and/or PA is responsible for the effects as this will be reported elsewhere. Improvements to wellbeing (FI) and mental health (AQoL-8D) were observed in the MedWalk group compared to the control group. The remaining AQoL-8D and GHQ-28 domains (and composite GHQ-28 score) were unchanged. Despite some regression over time, scores on all three outcome measures remained relatively consistent across the 12-month period, with no major decreases in participant responses, indicating a potential protective effect against decline in wellbeing, QoL and psychological health.

The improvements in wellbeing from baseline to 6 months were followed by a small regression back to baseline mean FI scores in the second 6 months of the intervention period. The improvements in the first 6 months were likely the result of the most intensive part of the intervention where visits with the dietitian and walking groups were most frequent. Interestingly, despite both groups reporting that their income met their needs at baseline, the MedDiet group had improvements to the Secure Flourish scores, likely the result of MedDiet food provision; however, this significant difference was not identified between 6 and 12 months and is unclear if it was due to the MedWalk intervention or due to extraneous factors.

Mood, psychological health and QoL are common outcomes of dietary and PA interventions and can be modified with short periods of exposure^(17,20,42)^. Considerable improvements have been reported in these outcomes; however, some populations include individuals with identified clinical conditions such as depression^(21,22)^, or high blood pressure (and other risk factors for CVD)^(18,19)^ where room for improvement is likely greater. The Australian HELFIMED RCT in 152 men and women aged 18–65 years with depression compared a MedDiet intervention with a social group comparator and found statistically significant improvements in all AQoL-8D domains (*P* < 0·001) except for ‘pain’ in both the MedDiet group and social group comparator^(43)^. These findings were sustained at the 6-month timepoint^(43)^. However, even at 6 months, where utility and domain scores were the highest for the HELFIMED social and MedDiet groups (lowest domain score = 0·47), these scores were far lower than our MedWalk and control group’s domain score at baseline, prior to intervention (lowest domain score = 0·70). The MedDairy^(19)^ and MedPork Australian RCT^(18)^ used the SF-36 instrument to measure QoL; however, improvements to the Role Emotional domain were only present in the MedPork study. The domains from the SF-36 are similar to those of the AQoL-8D, both of which are validated QoL measures^(44)^. Regarding QoL, the MedWalk group had significant improvements to the Mental Health domain only which is somewhat consistent with the study by Li *et al.* ^(45)^ who suggested that QoL improvements are mediated by changes in depression in a cohort of older Chinese adults^(45)^. According to AQoL-8D norms, the Mental Health domain score is at its peak during the 16–24 age group and 65–74 age group for males and the 55–64 age group and 65–74 age group for females^(35)^, the latter group is similar to participants recruited in the MedWalk trial. While the MedWalk participants in comparison to AQoL-8D norms are positioned at a stage of ‘peak mental health’, that is, the second peak of the ‘U’-shaped profile, improvements were still observed. Improvement in an already relatively healthy population has implications and raises the question of potential maximisation of impact for other populations and age groups with lower pre-existing QoL. Considering the HELFIMED population had self-reported depression prior to the intervention period, it is plausible that our MedWalk population had good pre-existing QoL with little room for improvement due to the high domain and index scores at baseline. Further, improvements in QoL from MedDiet studies appear to have a greater ability to modify emotional wellbeing and mental health domains compared to other domains such as Pain, Energy, Senses and Independent Living. For studies in ‘healthy’ older populations including the four-arm Australian 6-month LIILAC RCT, MedDiet and exercise group reported lower depression and anxiety stress scores (DASS) compared to the control group which is favourable^(20)^ and aligns with improvements to mental health observed by MedWalk participants across both the AQoL-8D Mental Health domain (and a trending reduction in the GHQ-28 Anxiety and Insomnia domain).

Although research on diet and its relationship to ‘flourishing’ is limited, evidence suggests potential benefits across various age groups and life stages, even though the direct link remains unclear. A correlational study by Conner *et al.* ^(4)^ in 405 adults from New Zealand aged 19·9 ± 1·6 years found improvements to ‘flourishing’ with higher consumption of fruit and vegetables. In the subsequent three-armed RCT in 171 adults aged 18–25 years, flourishing behaviours related to purposefulness and connectedness (*P* < 0·05) were reported in the two intervention arms that received fruit and vegetables as part of the intervention^(3)^. As our MedWalk population also received foods as part of the intervention, it is possible this influences flourishing and wellbeing, potentially through improving purpose. A follow-up study from the Edad con Salud project in 2397 men and women from Spain aged 21–101 years indicated higher MedDiet adherence was associated with better subjective wellbeing using the Cantril Self-Anchoring Striving Scale^(46)^. The Cantril scale or ‘ladder’ is a wellbeing assessment which determines if an individual is thriving, struggling or suffering. The scale has greater focus on future health and wellbeing as compared to the FI which focuses on current health and wellbeing and has a range of questions related to life satisfaction, meaning and purpose and mental and physical health^(47)^. Meanwhile, a study of 100 adults with overweight or obesity aged 19–57 years in Spain found no association with subjective wellbeing for fourteen-point Mediterranean Diet Adherence Screener (MEDAS) adherence, but fruit and vegetable intake were associated with increased life satisfaction using the eight-item positivity scale (8PS) and the happiness scale^(48)^. The 8PS has similar constructs to the FI with questions related to life satisfaction, meaning and purpose and social relationships but does not include questions that encompass mental and physical health or financial and material stability^(49)^. Interestingly, the LIILAC study identified a reduction in perceived wellness total scores (PWS) for the MedDiet and exercise group, which is not favourable as higher scores indicate greater perceived wellness. Values were controlled for a range of factors including baseline score, age, gender identity, education and baseline homocysteine concentrations^(20)^. Unlike the LIILAC cohort, our MedWalk group experienced improvements to flourishing and subjective wellbeing which similarly captures physical, spiritual, social and emotional dimensions and domains.

Nevertheless, there are a multitude of subjective assessments with few focusing on or measuring ‘flourishing’ and more general wellbeing which are important contributors to overall QoL and psychosocial health, especially in older adults. This is important considering flourishing behaviours, physical health, autonomy and life satisfaction may decline with increasing age. The MedDiet and PA have not been thoroughly explored in relation to these outcomes prior to the current research; however, links to individual components or food groups have been reported such as fruit and vegetables^(3,4)^. However, as we do not eat foods in isolation, it is important to consider the role of dietary patterns such as the MedDiet in flourishing.

We consider the limited impact in our MedWalk population, despite a large sample size to this population overall having good pre-existing wellbeing, QoL and psychological health. Our independent living population appears to be health conscious but otherwise relatively ‘healthy’ and high functioning according to the three measurements we have used. Further investigation is needed to determine how best to use combined MedDiet and walking interventions to target other areas of QoL and psychological health that were not modified after a 12-month trial.

### Limitations

The impact of COVID-19 on the MedWalk has been reported in the protocol paper^(32)^. In brief, trial delays, especially in Victoria where there were stricter mandated lockdowns compared to South Australia, led to withdrawal of participants and issues with recruitment^(32)^. The original sample size calculation was 364; however, only 161 participants were randomised, the majority of which were females which is consistent with other diet and lifestyle trials. Though we controlled for gender identity, it is possible the disproportionate gender identity split may have impacted results. Nevertheless, the trial was still adequately powered with the revised power calculations (80 % power, 7 % attrition and 5 % significance)^(32)^, but MedWalk was statistically powered for cognition as the primary outcome, not psychological health and wellbeing which may explain the limited relationship between the intervention and these outcomes.

Further, due to the delays in the MedWalk trial, the 24-month data collection timepoint was lost, meaning we were unable to follow up with participants after the intensive portion of the intervention had ended, to determine effects at a year post-intervention. This data is crucial to determine flow on health benefits and understand the sustainability of a MedDiet and lifestyle intervention in older Australian’s once a trial has ended, as seen in the South Australian MedLey trial follow-up, whereby participants were still adhering to certain parts of a MedDiet post-intervention^(50)^. While data from a small subset of South Australian participants has been documented elsewhere^(37)^, the restricted sample size substantially limits the ability to draw statistical inferences.

Lastly, when measuring psychological health and wellbeing, it can be complex to separate these concepts with many subjective measures capturing both. While psychological health is typically confined to mental and emotional domains, wellbeing represents a multidimensional concept incorporating life satisfaction alongside physical, social, emotional and spiritual health. Despite this distinction, the constructs are interrelated.

### Future directions

Future diet and lifestyle trials should continue to measure changes in QoL and psychosocial health as a primary and secondary outcome but should aim to recruit participants that may be at a higher risk of poor psychological health and wellbeing and have a more representative proportion of different gender identity.

Though psychosocial health surveys were completed at screening visits (Geriatric Depression Scale (GDS), DASS-21), it may be useful to use screening responses (i.e. the AQoL-8D, GHQ-28 or FI) as an exclusion factor or as part of the randomisation procedure, depending on the nature of the trial and other outcomes of interest. This may help to reduce the ceiling effect; however, this may limit recruitment from populations such as retirement villages and independent living facilities if there is a high pre-existing level of QoL and psychological health.

Further, future trials could also consider adopting a hybrid trial approach or mixed-methods approach and collect process data alongside clinical effectiveness data to help better understand the potential contextual factors at play and barriers to intervention success.

### Conclusions

In a relatively healthy older population, a 12-month MedDiet and walking intervention did not result in major changes to overall QoL and psychological health; however, it did lead to improvements in mental health. Further, wellbeing was improved during the first 6 months, and age, income meeting needs and self-reported health in some circumstances predicted improved scores across outcomes. These findings indicate improvements are achievable in a population who may already be considered to have adequate wellbeing, QoL and psychological health.

## Supporting information

Bracci et al. supplementary material 1 Bracci et al. supplementary material

Bracci et al. supplementary material 2 Bracci et al. supplementary material

## Supplementary material

For supplementary material accompanying this paper visit https://doi.org/10.1017/S1368980026102274
