# Supplementary material for: Wellbeing and quality of life secondary outcomes from a Mediterranean Diet and walking randomised controlled trial in older Australians
Source: Public Health Nutr. 2026 Mar 25;29(1):e78. doi: 10.1017/S1368980026102274 (PMC13087977; doi:10.1017/S1368980026102274)
Supplement: Bracci et al. supplementary material 2 — Bracci et al. supplementary material [file S1368980026102274sup002.docx]

**Additional File**

|  | MedWalk | | | Control | | |
| --- | --- | --- | --- | --- | --- | --- |
| Variable | **Baseline** | **6-months** | **12-months** | **Baseline** | **6-months** | **12-months** |
| Flourish Index |  |  |  |  |  |  |
| Total Flourish^a^ | 79.9 ± 1.59* | 82.5 ± 1.23* | 81.7 ± 1.55* | 80.3 ± 1.30 | 78.8 ± 1.35 | 79.8 ± 1.46 |
| Secure Flourish^b^ | 96.8 ± 1.76* | 99.4 ± 1.54* | 98.1 ± 2.00 | 97.9 ± 1.39 | 96.1 ± 1.49 | 96.9 ± 1.55 |
| AQoL-8D Domain^c^ |  |  |  |  |  |  |
| Independent living | 0.90 ± 0.01 |  | 0.90 ± 0.02 | 0.89 ± 0.01 |  | 0.87 ± 0.02 |
| Happiness | 0.84 ± 0.01 |  | 0.84 ± 0.01 | 0.84 ± 0.01 |  | 0.83 ± 0.01 |
| Mental Health | 0.70 ± 0.01 |  | 0.74 ± 0.02 | 0.72 ± 0.01***** |  | 0.71 ± 0.01***** |
| Relationships | 0.83 ± 0.01 |  | 0.84 ± 0.02 | 0.81 ± 0.02 |  | 0.82 ± 0.01 |
| Self-Worth | 0.87 ± 0.01 |  | 0.88 ± 0.01 | 0.87 ± 0.01 |  | 0.88 ± 0.01 |
| Pain | 0.80 ± 0.19 |  | 0.81 ± 0.02 | 0.79 ± 0.20 |  | 0.80 ± 0.02 |
| Senses | 0.86 ± 0.01 |  | 0.87 ± 0.01 | 0.87 ± 0.01 |  | 0.86 ± 0.01 |
| Coping | 0.84 ± 0.01 |  | 0.84 ± 0.01 | 0.83 ± 0.01 |  | 0.82 ± 0.01 |
| GHQ-28 Domain^e^ |  |  |  |  |  |  |
| Somatic | 4.50 ± 0.38 | 4.38 ± 0.41 | 4.41 ±0.42 | 4.61 ± 0.43 | 5.39 ± 0.50 | 5.30 ± 0.49 |
| Anxiety insomnia | 3.94 ± 0.37 | 3.36 ± 0.34 | 3.53 ± 0.36 | 3.55 ± 0.37 | 3.55 ±0.36 | 3.97 ±0.41 |
| Social dysfunction | 7.80 ± 0.27 | 7.63 ± 0.29 | 7.85 ± 0.31 | 8.34 ± 0.32 | 8.52 ± 0.32 | 8.26 ± 0.31 |
| Severe depression | 1.56 ± 0.13 | 1.40 ± 0.12 | 1.48 ± 0.13 | 1.43 ± 0.13 | 1.49 ± 0.14 | 1.45 ± 0.13 |
| Composite GHQ score | 14.5 ±0.91 | 13.5 ± 0.91 | 14.0 ± 0.95 | 14.5 ± 0.99 | 15.6 ±1.07 | 15.6 ± 1.06 |

**Table A1.** Raw mean Flourishing Index, AQoL-8D and GHQ-28 scores at baseline, 6-months, and 12-months (n=161).

All results are mean ± SEM; ^a^maximum score = 100; ^b^maximum score = 120; a higher score indicates greater wellbeing; AQoL-8D^c^ = Assessment of Quality of Life 8 dimensions with scores ranging from -0.04 (worse than death) to 1.0 (full health); high AQoL-8D scores are positive; higher scores on the GHQ-28 = ^e^greater psychological distress and are negative. * indicates statistically significant change between the two timepoints (p<.05).

**Table A2.** Tests of Between-Subjects group effects for all variables across change score general linear model analyses for Flourishing Index and AQoL-8D domains when controlling for state, gender, income, self-reported health, age, and education years.

| Dependent Variable | Type III Sum of Squares | Mean Square | F | p-value | Partial Eta Squared |
| --- | --- | --- | --- | --- | --- |
| Total Flourish (baseline, 6, 12-months) |  |  |  |  |  |
| Baseline to 12-months |  |  |  |  |  |
| Group | 19.524 | 19.524 | .294 | .589 | .002 |
| State | 182.070 | 182.070 | 2.738 | .100 | .022 |
| Gender | 6.375 | 6.375 | .096 | .757 | .001 |
| Income_binary | 60.106 | 60.106 | .904 | .344 | .007 |
| Health_binary | 87.446 | 87.446 | 1.315 | .254 | .010 |
| Age | 51.744 | 51.744 | .778 | .379 | .006 |
| Education_years | 150.340 | 150.340 | 2.261 | .135 | .018 |
| 6-months to 12-months |  |  |  |  |  |
| Group | 250.109 | 250.109 | 4.322 | **.040*** | .034 |
| State | 270.077 | 270.077 | 4.668 | .**033*** | .036 |
| Gender | 48.036 | 48.036 | .830 | .364 | .007 |
| Income_binary | 13.218 | 13.218 | .228 | .634 | .002 |
| Health_binary | .237 | .237 | .004 | .949 | .000 |
| Age | 7.263 | 7.263 | .126 | .724 | .001 |
| Education_years | 79.371 | 79.371 | 1.372 | .244 | .011 |
| Baseline to 6-months |  |  |  |  |  |
| Group | 516.109 | 516.109 | 9.303 | **.003**** | .071 |
| State | 13.879 | 13.879 | .250 | .618 | .002 |
| Gender | 53.430 | 53.430 | .963 | .328 | .008 |
| Income_binary | 222.290 | 222.290 | 4.007 | **.048*** | .032 |
| Health_binary | 31.287 | 31.287 | .564 | .454 | .005 |
| Age | 44.702 | 44.702 | .806 | .371 | .007 |
| Education_years | 21.587 | 21.587 | .389 | .534 | .003 |
| Secure Flourish (baseline, 6, 12-months) |  |  |  |  |  |
| Baseline to 12-months |  |  |  |  |  |
| Group | 16.038 | 16.038 | .212 | .646 | .002 |
| State | 170.155 | 170.155 | 2.245 | .137 | .018 |
| Gender | 4.855 | 4.855 | .064 | .801 | .001 |
| Income_binary | 78.248 | 78.248 | 1.032 | .312 | .008 |
| Health_binary | 125.694 | 125.694 | 1.658 | .200 | .013 |
| Age | 13.517 | 13.517 | .178 | .674 | .001 |
| Education_years | 205.798 | 205.798 | 2.715 | .102 | .021 |
| 6-months to 12-months |  |  |  |  |  |
| Group | 246.488 | 246.488 | 2.676 | .104 | .021 |
| State | 193.342 | 193.342 | 2.099 | .150 | .017 |
| Gender | 240.461 | 240.461 | 2.611 | .109 | .021 |
| Income_binary | 16.004 | 16.004 | .174 | .678 | .001 |
| Health_binary | 14.968 | 14.968 | .163 | .688 | .001 |
| Age | .093 | .093 | .001 | .975 | .000 |
| Education_years | 119.581 | 119.581 | 1.298 | .257 | .010 |
| Baseline to 6-months |  |  |  |  |  |
| Group | 577.947 | 577.947 | 7.031 | .**009**** | .054 |
| State | 12.511 | 12.511 | .152 | .697 | .001 |
| Gender | 99.877 | 99.877 | 1.215 | .273 | .010 |
| Income_binary | 308.133 | 308.133 | 3.748 | .055 | .030 |
| Health_binary | 98.602 | 98.602 | 1.199 | .276 | .010 |
| Age | 38.289 | 38.289 | .466 | .496 | .004 |
| Education_years | 10.462 | 10.462 | .127 | .722 | .001 |
| AQoL-8D Domain |  |  |  |  |  |
| Independent living Baseline to 12-months |  |  |  |  |  |
| Group | .018 | .018 | 2.362 | .127 | .019 |
| State | .006 | .006 | .767 | .383 | .006 |
| Gender | .053 | .053 | 6.837 | .**010*** | .052 |
| Income_binary | .011 | .011 | 1.374 | .243 | .011 |
| Health_binary | .001 | .001 | .068 | .795 | .001 |
| Age | .015 | .015 | 1.923 | .168 | .015 |
| Education_years | .010 | .010 | 1.331 | .251 | .011 |
| Happiness Baseline to 12-months |  |  |  |  |  |
| Group | .001 | .001 | .302 | .584 | .002 |
| State | .008 | .008 | 1.757 | .187 | .014 |
| Gender | .006 | .006 | 1.216 | .272 | .010 |
| Income_binary | .004 | .004 | .793 | .375 | .006 |
| Health_binary | .008 | .008 | 1.794 | .183 | .014 |
| Age | .012 | .012 | 2.663 | .105 | .021 |
| Education_years | .003 | .003 | .620 | .432 | .005 |
| Mental Health Baseline to 12-months |  |  |  |  |  |
| Group | .024 | .024 | 3.970 | **.048*** | .031 |
| State | .006 | .006 | .962 | .329 | .008 |
| Gender | .001 | .001 | .150 | .700 | .001 |
| Income_binary | .004 | .004 | .617 | .434 | .005 |
| Health_binary | .005 | .005 | .752 | .387 | .006 |
| Age | .003 | .003 | .536 | .465 | .004 |
| Education_years | .002 | .002 | .303 | .583 | .002 |
| Relationships Baseline to 12-months |  |  |  |  |  |
| Group | .000 | .000 | .011 | .915 | .000 |
| State | .002 | .002 | .246 | .621 | .002 |
| Gender | .000 | .000 | .044 | .834 | .000 |
| Income_binary | .048 | .048 | 4.992 | **.027*** | .038 |
| Health_binary | .017 | .017 | 1.728 | .191 | .014 |
| Age | 4.065E-5 | 4.065E-5 | .004 | .948 | .000 |
| Education_years | .005 | .005 | .565 | .454 | .004 |
| Pain Baseline to 12-months |  |  |  |  |  |
| Group | .010 | .010 | .408 | .524 | .003 |
| State | .001 | .001 | .036 | .850 | .000 |
| Gender | .022 | .022 | .851 | .358 | .007 |
| Income_binary | 6.434E-5 | 6.434E-5 | .003 | .960 | .000 |
| Health_binary | .030 | .030 | 1.180 | .280 | .009 |
| Age | .048 | .048 | 1.901 | .170 | .015 |
| Education_years | .014 | .014 | .555 | .458 | .004 |
| Senses Baseline to 12-months |  |  |  |  |  |
| Group | .007 | .007 | .862 | .355 | .007 |
| State | .013 | .013 | 1.601 | .208 | .013 |
| Gender | .004 | .004 | .498 | .482 | .004 |
| Income_binary | .000 | .000 | .014 | .906 | .000 |
| Health_binary | .000 | .000 | .028 | .867 | .000 |
| Age | .084 | .084 | 10.187 | **.002**** | .075 |
| Education_years | .000 | .000 | .048 | .826 | .000 |
| Coping Baseline to 12-months |  |  |  |  |  |
| Group | .000 | .000 | .012 | .912 | .000 |
| State | .004 | .004 | .475 | .492 | .004 |
| Gender | .002 | .002 | .186 | .667 | .001 |
| Income_binary | .001 | .001 | .167 | .684 | .001 |
| Health_binary | .037 | .037 | 4.280 | **.041*** | .033 |
| Age | .002 | .002 | .263 | .609 | .002 |
| Education_years | .002 | .002 | .243 | .623 | .002 |

P<.05*, p<.01**, p<.001***;

Please note: Each time period is a separate GLM i.e., baseline to 12-months, 6-months to 12-months and baseline to 6-months.

**Table A3.** Parameter estimates for all variables across change score general linear model analyses for Flourishing Index and AQoL-8D domains when controlling for state, gender, income, self-reported health, age, and education years.

| Parameter | B | Std. Error | p-value | 95% Confidence Interval | | Partial Eta Squared |
| --- | --- | --- | --- | --- | --- | --- |
|  |  |  |  | **Lower Bound** | **Upper Bound** |  |
| Total Flourish |  |  |  |  |  |  |
| Baseline to 12-months |  |  |  |  |  |  |
| [Group=1.00] | .805 | 1.486 | .589 | -2.136 | 3.747 | .002 |
| [State=1.00] | 2.604 | 1.574 | .100 | -.511 | 5.719 | .022 |
| [Gender=1.00] | .506 | 1.636 | .757 | -2.731 | 3.744 | .001 |
| [Income_binary=.00] | -1.511 | 1.589 | .344 | -4.655 | 1.634 | .007 |
| [Health_binary=.00] | 2.656 | 2.316 | .254 | -1.928 | 7.240 | .010 |
| Age | .115 | .130 | .379 | -.143 | .372 | .006 |
| Education_years | .322 | .214 | .135 | -.102 | .746 | .018 |
| 6-months to 12-months |  |  |  |  |  |  |
| [Group=1.00] | -2.902 | 1.396 | **.040*** | -5.664 | -.139 | .034 |
| [State=1.00] | 3.225 | 1.493 | .**033*** | .270 | 6.179 | .036 |
| [Gender=1.00] | -1.401 | 1.537 | .364 | -4.444 | 1.642 | .007 |
| [Gender=2.00] | 0^a^ | . | . | . | . | . |
| [Income_binary=.00] | .708 | 1.481 | .634 | -2.224 | 3.640 | .002 |
| [Health_binary=.00] | -.138 | 2.147 | .949 | -4.387 | 4.112 | .000 |
| Age | .044 | .124 | .724 | -.201 | .289 | .001 |
| Education_years | .245 | .209 | .244 | -.169 | .659 | .011 |
| Baseline to 6-months |  |  |  |  |  |  |
| [Group=1.00] | 4.154 | 1.362 | **.003**** | 1.458 | 6.850 | .071 |
| [State=1.00] | -.732 | 1.463 | .618 | -3.629 | 2.165 | .002 |
| [Gender=1.00] | 1.483 | 1.511 | .328 | -1.508 | 4.474 | .008 |
| [Income_binary=.00] | -2.885 | 1.442 | **.048*** | -5.739 | -.032 | .032 |
| [Health_binary=.00] | 1.544 | 2.056 | .454 | -2.526 | 5.613 | .005 |
| Age | .110 | .123 | .371 | -.133 | .353 | .007 |
| Education_years | .128 | .205 | .534 | -.279 | .535 | .003 |
| Secure Flourish |  |  |  |  |  |  |
| Baseline to 12-months |  |  |  |  |  |  |
| [Group=1.00] | .730 | 1.587 | .646 | -2.411 | 3.871 | .002 |
| [State=1.00] | 2.517 | 1.680 | .137 | -.808 | 5.843 | .018 |
| [Gender=1.00] | -.442 | 1.746 | .801 | -3.899 | 3.015 | .001 |
| [Income_binary=.00] | -1.724 | 1.696 | .312 | -5.081 | 1.634 | .008 |
| [Health_binary=.00] | 3.184 | 2.473 | .200 | -1.710 | 8.079 | .013 |
| Age | .059 | .139 | .674 | -.216 | .333 | .001 |
| Education_years | .377 | .229 | .102 | -.076 | .829 | .021 |
| 6-months to 12months |  |  |  |  |  |  |
| [Group=1.00] | -2.881 | 1.761 | .104 | -6.366 | .605 | .021 |
| [State=1.00] | 2.728 | 1.883 | .150 | -.999 | 6.456 | .017 |
| [Gender=1.00] | -3.134 | 1.940 | .109 | -6.974 | .705 | .021 |
| [Income_binary=.00] | .779 | 1.869 | .678 | -2.920 | 4.478 | .001 |
| [Health_binary=.00] | -1.092 | 2.709 | .688 | -6.454 | 4.270 | .001 |
| Age | -.005 | .156 | .975 | -.314 | .304 | .000 |
| Education_years | .301 | .264 | .257 | -.222 | .824 | .010 |
| Baseline to 6-months |  |  |  |  |  |  |
| [Group=1.00] | 4.396 | 1.658 | **.009**** | 1.114 | 7.677 | .054 |
| [State=1.00] | -.695 | 1.781 | .697 | -4.221 | 2.831 | .001 |
| [Gender=1.00] | 2.027 | 1.839 | .273 | -1.614 | 5.669 | .010 |
| [Income_binary=.00] | -3.397 | 1.755 | .055 | -6.871 | .076 | .030 |
| [Health_binary=.00] | 2.740 | 2.502 | .276 | -2.213 | 7.693 | .010 |
| Age | .102 | .149 | .496 | -.194 | .397 | .004 |
| Education_years | .089 | .250 | .722 | -.406 | .584 | .001 |
| AQoL-8D Domain |  |  |  |  |  |  |
| Independent living Baseline to 12-months |  |  |  |  |  |  |
| [Group=1.00] | .024 | .016 | .127 | -.007 | .056 | .019 |
| [State=1.00] | .015 | .017 | .383 | -.019 | .048 | .006 |
| [Gender=1.00] | -.046 | .018 | **.010*** | -.081 | -.011 | .052 |
| [Income_binary=.00] | -.020 | .017 | .243 | -.054 | .014 | .011 |
| [Health_binary=.00] | .006 | .024 | .795 | -.042 | .054 | .001 |
| Age | -.002 | .001 | .168 | -.005 | .001 | .015 |
| Education_years | .003 | .002 | .251 | -.002 | .007 | .011 |
| Happiness Baseline to 12-months |  |  |  |  |  |  |
| [Group=1.00] | -.007 | .012 | .584 | -.031 | .018 | .002 |
| [State=1.00] | .017 | .013 | .187 | -.009 | .043 | .014 |
| [Gender=1.00] | .015 | .014 | .272 | -.012 | .042 | .010 |
| [Income_binary=.00] | -.012 | .013 | .375 | -.038 | .014 | .006 |
| [Health_binary=.00] | .025 | .019 | .183 | -.012 | .062 | .014 |
| Age | .002 | .001 | .105 | .000 | .004 | .021 |
| Education_years | .001 | .002 | .432 | -.002 | .005 | .005 |
| Mental Health Baseline to 12-months |  |  |  |  |  |  |
| [Group=1.00] | .028 | .014 | **.048*** | .000 | .056 | .031 |
| [State=1.00] | .015 | .015 | .329 | -.015 | .045 | .008 |
| [Gender=1.00] | -.006 | .016 | .700 | -.037 | .025 | .001 |
| [Income_binary=.00] | .012 | .015 | .434 | -.018 | .042 | .005 |
| [Health_binary=.00] | .019 | .022 | .387 | -.024 | .061 | .006 |
| Age | .001 | .001 | .465 | -.002 | .003 | .004 |
| Education_years | -.001 | .002 | .583 | -.005 | .003 | .002 |
| Relationships Baseline to 12-months |  |  |  |  |  |  |
| [Group=1.00] | .002 | .018 | .915 | -.033 | .037 | .000 |
| [State=1.00] | -.009 | .019 | .621 | -.047 | .028 | .002 |
| [Gender=1.00] | -.004 | .020 | .834 | -.043 | .035 | .000 |
| [Income_binary=.00] | .043 | .019 | .**027*** | .005 | .080 | .038 |
| [Health_binary=.00] | .036 | .027 | .191 | -.018 | .089 | .014 |
| Age | .000 | .002 | .948 | -.003 | .003 | .000 |
| Education_years | -.002 | .003 | .454 | -.007 | .003 | .004 |
| Self-Worth Baseline to 12-months |  |  |  |  |  |  |
| [Group=1.00] | .001 | .014 | .956 | -.027 | .028 | .000 |
| [State=1.00] | .002 | .015 | .889 | -.027 | .031 | .000 |
| [Gender=1.00] | -.007 | .015 | .643 | -.038 | .023 | .002 |
| [Income_binary=.00] | -.002 | .015 | .914 | -.031 | .028 | .000 |
| [Health_binary=.00] | .009 | .021 | .669 | -.033 | .051 | .001 |
| Age | .002 | .001 | .141 | -.001 | .004 | .017 |
| Education_years | .001 | .002 | .678 | -.003 | .005 | .001 |
| Pain Baseline to 12-months |  |  |  |  |  |  |
| [Group=1.00] | .018 | .029 | .524 | -.039 | .076 | .003 |
| [State=1.00] | .006 | .031 | .850 | -.055 | .067 | .000 |
| [Gender=1.00] | -.030 | .032 | .358 | -.094 | .034 | .007 |
| [Income_binary=.00] | .002 | .031 | .960 | -.060 | .063 | .000 |
| [Health_binary=.00] | .048 | .044 | .280 | -.039 | .134 | .009 |
| Age | .003 | .003 | .170 | -.002 | .009 | .015 |
| Education_years | -.003 | .004 | .458 | -.011 | .005 | .004 |
| Senses Baseline to 12-months |  |  |  |  |  |  |
| [Group=1.00] | .015 | .016 | .355 | -.017 | .048 | .007 |
| [State=1.00] | -.022 | .017 | .208 | -.057 | .012 | .013 |
| [Gender=1.00] | -.013 | .018 | .482 | -.049 | .023 | .004 |
| [Income_binary=.00] | -.002 | .018 | .906 | -.037 | .033 | .000 |
| [Health_binary=.00] | -.004 | .025 | .867 | -.054 | .045 | .000 |
| Age | .005 | .001 | **.002**** | .002 | .007 | .075 |
| Education_years | .001 | .002 | .826 | -.004 | .005 | .000 |
| Coping Baseline to 12-months |  |  |  |  |  |  |
| [Group=1.00] | .002 | .017 | .912 | -.031 | .035 | .000 |
| [State=1.00] | .012 | .018 | .492 | -.023 | .048 | .004 |
| [Gender=1.00] | .008 | .019 | .667 | -.029 | .045 | .001 |
| [Income_binary=.00] | -.007 | .018 | .684 | -.043 | .028 | .001 |
| [Health_binary=.00] | .053 | .026 | **.041*** | .002 | .103 | .033 |
| Age | -.001 | .001 | .609 | -.004 | .002 | .002 |
| Education_years | .001 | .002 | .623 | -.004 | .006 | .002 |

Group 1 = MedWalk as compared to Group 2 = Control State=1.00 = South Australia as compared to State=2.00 = Victoria; Gender =1.00 = Female as compared to Gender = 2.00 = Male; Income_binary = .00 = always have enough income compared to 1.00 = do not always have enough income; Health_binary = .00 = very good, good compared to 1.00 = average, poor, or very poor health

Please note: Each time period is a separate GLM i.e., baseline to 12-months, 6-months to 12-months and baseline to 6-months.

**Table A4.** Composite GHQ-28 and domain parameter and coefficients for all variables when controlling for state, age, income, self-reported health at baseline, gender and education years

| Model Term | Coefficient | Std. Error | Sig. | 95% Confidence Interval | |
| --- | --- | --- | --- | --- | --- |
|  |  |  |  | **Lower** | **Upper** |
| Somatic |  |  |  |  |  |
| Time=1 | -.137 | .077 | .074 | -.288 | .014 |
| Time=2 | .017 | .076 | .824 | -.133 | .166 |
| Group=1 | -.187 | .108 | .084 | -.400 | .026 |
| State=1 | -.125 | .091 | .170 | -.303 | .054 |
| Age | .002 | .007 | .796 | -.013 | .016 |
| Income_binary=0 | -.046 | .089 | .604 | -.222 | .129 |
| Health_binary=0 | -.469 | .123 | **<.001***** | -.710 | -.227 |
| Gender=1 | .097 | .094 | .302 | -.088 | .283 |
| Education_years | .008 | .012 | .531 | -.017 | .032 |
| [Time=1]*[Group=1] | .166 | .109 | .130 | -.049 | .380 |
| [Time=2]*[Group=1] | -.020 | .111 | .855 | -.237 | .19 |
| Anxiety Insomnia |  |  |  |  |  |
| Time=1 | -.106 | .073 | .144 | -.249 | .036 |
| Time=2 | -.110 | .072 | .124 | -.251 | .030 |
| Group=1 | -.120 | .117 | .306 | -.350 | .110 |
| State=1 | -.197 | .105 | .060 | -.403 | -.009 |
| Age | .007 | .009 | .411 | -.010 | .024 |
| Income_binary=0 | -.091 | .103 | .377 | -.293 | .111 |
| Health_binary=0 | -.343 | .141 | **.015*** | -.620 | -.067 |
| Gender=1 | .153 | .109 | .160 | -.061 | .367 |
| Education_years | .011 | .014 | .454 | -.018 | .039 |
| [Time=1]*[Group=1] | .227 | .103 | **.028*** | .024 | .430 |
| [Time=2]*[Group=1] | .065 | .104 | .534 | -.140 | .270 |
| Social Dysfunction |  |  |  |  |  |
| Time=1 | .011 | .033 | .748 | -.054 | .076 |
| Time=2 | .031 | .033 | .347 | -.034 | .096 |
| Group=1 | -.052 | .045 | .250 | -.139 | .036 |
| State=1 | -.059 | .036 | .106 | -.130 | .013 |
| Age | .010 | .003 | **.001**** | .004 | .016 |
| Income_binary=0 | .057 | .036 | .110 | -.013 | .128 |
| Health_binary=0 | -.127 | .049 | **.011*** | -.224 | -.029 |
| Gender=1 | .007 | .038 | .852 | -.067 | .082 |
| Education_years | .007 | .005 | .172 | -.003 | .017 |
| [Time=1]*[Group=1] | -.015 | .047 | .755 | -.107 | .078 |
| [Time=2]*[Group=1] | -.059 | .048 | .220 | -.153 | .035 |
| Severe Depression |  |  |  |  |  |
| Time=1 | -.018 | .061 | .771 | -.138 | .102 |
| Time=2 | .026 | .060 | .667 | -.093 | .145 |
| Group=1 | .016 | .102 | .873 | -.185 | .218 |
| State=1 | -.179 | .093 | .055 | -.361 | .004 |
| Age | .006 | .008 | .461 | -.009 | .020 |
| Income_binary=0 | .116 | .091 | .204 | -.063 | .296 |
| Health_binary=0 | -.340 | .125 | **.007**** | -.585 | -.094 |
| Gender=1 | .022 | .097 | .823 | -.168 | .212 |
| Education_years | .003 | .013 | .803 | -.022 | .028 |
| [Time=1]*[Group=1] | .077 | .087 | .375 | -.094 | .248 |
| [Time=2]*[Group=1] | -.081 | .088 | .356 | -.253 | .091 |
| Composite GHQ-28 |  |  |  |  |  |
| Time=1 | -.071 | .050 | .161 | -.169 | .028 |
| Time=2 | .005 | .050 | .916 | -.092 | .103 |
| Group=1 | -.108 | .078 | .169 | -.261 | .046 |
| State=1 | -.148 | .069 | **.032*** | -.284 | -.013 |
| Age | .010 | .006 | .077 | -.001 | .021 |
| Income_binary=0 | -.003 | .068 | .970 | -.136 | .131 |
| Health_binary=0 | -.371 | .093 | **<.001***** | -.554 | -.189 |
| Gender=1 | .080 | .072 | .263 | -.061 | .221 |
| Education_years | .010 | .100 | .275 | -.008 | .029 |
| [Time=1]*[Group=1] | .113 | .072 | .114 | -.027 | .254 |
| [Time=2]*[Group=1] | -.036 | .072 | .618 | -.178 | .106 |

State 1= South Australia; State 2= Victoria; [Time =1] = baseline; [Time = 2]= 6-months; [Time=3]= 12-months; Group 1 = MedWalk; Group 2 =Control; Time 1 and 2 compared to time 3; [Time=1]*[Group=1] compared to [Time=3]*[Group =2]; p<0.05*; p<0.01**; p<0.001***; Gender =1.00 = Female as compared to Gender = 2.00 = Male; Income_binary = .00 = always have enough income compared to 1.00 = do not always have enough income; Health_binary = .00 = very good or good compared to 1.00 = average, poor or very poor health
